# Association of Shoot and Root Responses to Water Deficit in Young Faba Bean (*Vicia faba L.*) Plants

**DOI:** 10.3389/fpls.2019.01063

**Published:** 2019-09-04

**Authors:** Kiflemariam Y. Belachew, Kerstin A. Nagel, Hendrik Poorter, Frederick L. Stoddard

**Affiliations:** ^1^Department of Agricultural Sciences, Viikki Plant Science Centre, University of Helsinki, Helsinki, Finland; ^2^Department of Plant Sciences, Bahir Dar University, Bahir Dar, Ethiopia; ^3^IBG-2: Plant Sciences, Forschungszentrum Jülich GmbH, Jülich, Germany; ^4^Department of Biological Sciences, Macquarie University, North Ryde, NSW, Australia

**Keywords:** drought avoidance, drought phenotyping, shoot traits, root traits, abiotic stress, water use efficiency

## Abstract

Water deficit may occur at any stage of plant growth, with any intensity and duration. Phenotypic acclimation and the mechanism of adaptation vary with the evolutionary background of germplasm accessions and their stage of growth. Faba bean is considered sensitive to various kinds of drought. Hence, we conducted a greenhouse experiment in rhizotrons under contrasting watering regimes to explore shoot and root traits and drought avoidance mechanisms in young faba bean plants. Eight accessions were investigated for shoot and root morphological and physiological responses in two watering conditions with four replications. Pre-germinated seedlings were transplanted into rhizotron boxes filled with either air-dried or moist peat. The water-limited plants received 50-ml water at transplanting and another 50-ml water 4 days later, then no water was given until the end of the experimental period, 24 days after transplanting. The well-watered plants received 100 ml of water every 12 h throughout the experimental period. Root, stem, and leaf dry mass, their mass fractions, their dry matter contents, apparent specific root length and density, stomatal conductance, SPAD value, and Fv/Fm were recorded. Water deficit resulted in 3–4-fold reductions in shoot biomass, root biomass, and stomatal conductance along with 1.2–1.4-fold increases in leaf and stem dry matter content and SPAD values. Total dry mass and apparent root length density showed accession by treatment interactions. Accessions DS70622, DS11320, and ILB938/2 shared relatively high values of total dry mass and low values of stomatal conductance under water deficit but differed in root distribution parameters. In both treatments, DS70622 was characterized by finer roots that were distributed in both depth and width, whereas DS11320 and ILB938/2 produced less densely growing, thicker roots. French accession Mélodie/2 was susceptible to drought in the vegetative phase, in contrast to previous results from the flowering phase, showing the importance of timing of drought stress on the measured response. Syrian accession DS70622 explored the maximum root volume and maintained its dry matter production, with the difference from the other accessions being particularly large in the water-limited treatment, so it is a valuable source of traits for avoiding transient drought.

## Introduction

Drought is a critical problem in worldwide grain legume production (Stoddard et al., 2006; Farooq et al., 2017; Ye et al., 2018). Limitation of soil moisture can occur at any stage of crop growth, and the traits that respond to the changing soil–atmosphere environment vary with the stage of development (Coleman et al., 1994; Blum, 1996; Poorter et al., 2010; Passioura, 2012; Tuberosa, 2012; Gosa et al., 2018). Differences in crop response between climate-controlled growth facilities and field conditions often hamper the identification of target traits for the stress in question (Poorter et al., 2016). Consequently, crop improvement programs find it a challenge to identify suitable traits for drought tolerance in specific germplasm, at a particular stage of growth and appropriate for local growing condition (Halperin et al., 2016; Negin and Moshelion, 2017). Drought response strategies have been classified into escape by earliness of seed maturation, avoidance by maintenance of turgor through increased water-finding ability and appropriate stomatal activity, and tolerance by biochemical means such as detoxification of reactive oxygen species (Levitt, 1980).

Faba bean (*Vicia faba* L.) is an important crop in semi-arid regions around the world, but it is considered to be sensitive to moisture stress (Loss et al., 1997a). Escape from terminal drought is used in many semi-arid environments (Loss et al., 1997b; Link et al., 2008), and osmotic adjustment (OA) has not yet been shown (Khan et al., 2010; Duc et al., 2015), but avoidance of transient drought is challenging to investigate because of the range of severities and timings that it can take. Wide variation in shoot and root responses to water deficit has been found at the flowering (Khan et al., 2007; Khazaei et al., 2013a; Khazaei et al., 2013b) and vegetative stages (Belachew et al., 2018). Young faba bean plants exhibiting deeper and wider root system showed enhanced potential for drought avoidance by water scavenging (Belachew et al., 2018). In a close relative, lentil (*Lens culinaris* Medik.), genotypic variation was found in the ability of juvenile plants to maintain growth in water deficit conditions (Singh et al., 2013), and root mass was positively correlated with chlorophyll content and shoot mass in response to early-stage drought (Idrissi et al., 2015). Different stages of plant growth may react to water deficit differently, as shown in wheat (Passioura, 2012) and chickpea (*Cicer arietinum* L.) (Purushothaman et al., 2016).

Canopy temperature is widely variable in faba bean germplasm (Khazaei et al., 2013a), and its depression is strongly and positively correlated with higher stomatal conductance and greater carbon isotope discrimination (Δ^13^C; Khan et al., 2007). Measurement of maximum quantum yield of photosystem II (Fv/Fm) was found to be a useful tool to discriminate faba bean genotypes against cold and heat stresses (Zhou et al., 2018), both of which are associated with dehydration and oxidative stress (Gogoi et al., 2018). Other rapidly screened indirect indicators of drought tolerance positively correlating with yield shown in other species include chlorophyll stability in peanut (*Arachis hypogaea* L.) (Arunyanark et al., 2008), higher chlorophyll content and Fv/Fm in barley (Li et al., 2006), stem length in lentil (Sarker et al., 2005), and stem greenness in common bean (*Phaseolus vulgaris* L.) seedlings (Mukeshimana et al., 2014).

When seeking sources of stress resistance, a first step often comprises investigating a relatively large number of morphological and physiological traits on a wide range of germplasm (Khazaei et al., 2013a; Belachew and Stoddard, 2017; Belachew et al., 2018), followed by focus on specific traits in a reduced number of accessions to identify potential mechanisms of action and potential for combining different mechanisms (Khazaei et al., 2013b; Purushothaman et al., 2016). Deeper roots may confer drought avoidance (Matsui, and Singh 2003; Zhao et al. 2017; Belachew et al., 2018; Ye et al., 2018), while lower stomatal conductance under soil moisture deficiency will have an advantage when there is high transpiration demand due to high vapor pressure deficit (Reynolds and Trethowan, 2007). On the other hand, higher stomatal conductance is positively correlated with higher biomass or yield gain (Blum, 2015; Gosa et al., 2018), as stomatal closure hinders carbon assimilation. Although early closure of stomata is one of the mechanisms by which faba bean deals with drought stress (Link et al., 2008), it often arises from the inability of roots to access moisture from the soil (Blum, 2009; Tuberosa, 2012). Hence, combinations of traits are important to improve the yield of faba bean and other crops for drought-affected areas (Khan et al., 2007; Krishnamurthy et al., 2010).

Water deficit at an early stage of growth can occur in any environment, and the strategy of escape by earliness is not available, so sources of avoidance and/or tolerance are needed. For these reasons, we set out to investigate the drought avoidance mechanisms of a set of faba bean accessions in the vegetative phase. Our hypothesis was that drought avoidance is based on a combination of leaf gas exchange and exploitation of soil water, so evaluation of both roots and shoots was needed.

## Material and Methods

Eight accessions (see **Table 1** for country of origin) were subjected to root and shoot phenotyping in an experiment carried out at the Research Centre Jülich, Germany in winter 2017, as described previously (Belachew et al., 2018). Briefly, seeds were germinated in Petri dishes, and uniform and healthy-looking seedlings were transplanted into an automated root and shoot phenotyping platform GROWSCREEN-Rhizo (using rhizotrons of 90 * 70 * 5 cm) (Nagel et al., 2012) in the greenhouse. Two treatments were applied in four biological replicates. Rhizotrons for the well-watered treatment were filled with the peat medium as received with moisture content of 66.3%, but those for the water-limited treatment were filled with air-dried peat to 40% moisture content. The well-watered plants automatically received 100 ml of water every 12 h until the end of the treatment period, whereas in the water-deficit treatment, plants received 50 ml of water at transplanting and 50 ml 4 days later. During the experiment, plants were grown at 20.9°C air temperature and 58% air humidity, and 22.6°C average growing media temperature, in a photoperiod of 15 h light and 9 h dark, daily light integral of 4.3 mol.m^-2^.d^-1^. Details about the growth media, treatments, experimental design, and plant management were given in Belachew et al. (2018). Shoot and root morphological and physiological data that were recorded 15 to 19 days after treatment (DAT) are reported in this paper.

**Table 1:** Biomass production and apparent specific root length and density of eight faba bean accessions in well-watered and water-limited conditions 28 days after seed imbibition, four biological replicates. ns is not significant, SE is standard error of the mean, LSD is least significant difference.

Accession	Country of origin	Shoot dry mass (g)		Apparent specific root length (m/g)		Apparent root length density (cm/cm^3^)	
		Well watered	Water limited	Well watered	Water limited	Well watered	Water limited
DS11202	Jordan	0.72	0.13	22.4	17.6	0.074	0.075
DS11320	Macedonia	1.19	0.38	14.4	8.0	0.091	0.061
DS70622	Syria	1.90	0.42	19.2	24.1	0.104	0.110
DS74573	Russia	1.16	0.24	19.9	18.6	0.082	0.079
EH06006-6	Ethiopia	1.91	0.56	16.1	16.0	0.062	0.100
ILB938/2	Ecuador	1.34	0.34	11.4	7.9	0.076	0.081
Mélodie/2	France	0.43	0.15	13.0	13.9	0.053	0.063
WS99501	China	0.91	0.08	14.5	8.7	0.104	0.052
Mean		1.19	0.28	16.3	14.4	0.081	0.077
SE (accessions)		0.14		1.8		0.013	
LSD (5%)		0.39		5.0		0.037	
SE (treatments)		0.02		0.9		0.005	
LSD (5%)		0.07		ns		ns	
**P-value**							
Treatment		<0.001		0.468		0.580	
Accession		<0.001		<0.001		0.052	
Treatment x Accession		0.002		0.313		0.050	

### Data Collected

Leaves were counted at 2, 9, and 16 DAT and during plant harvest (19 DAT). Stomatal conductance was measured between 11:00 and 13:00 local time on each plant using a Leaf Porometer (Decagon Devices, Inc, Pullman, WA, U.S.A.) at 15 DAT. Leaf chlorophyll concentration was estimated from two leaves per plant, and the average of the two was recorded using a SPAD-502 (Minolta Camera Co, Ltd, Japan) meter according to the manufacturer’s instructions at 15 DAT.

Chlorophyll fluorescence measurements were taken using a pulse-amplitude-modulated fluorometer (MINI-PAM, Heinz Walz GmBH, 91090 Effeltrich, Germany), according to the manufacturer’s instructions, to determine the effects of drought on electron transport system of photosystem II. The maximum quantum yield of photosystem II (Fv/Fm) and effective quantum yield (EQY) were taken from the youngest fully expanded leaf after 25 min of dark adaptation (using leaf clips, DLC-8, Heinz Walz GmbH, Eiffeltrich, Germany) between 07:00 and 08:00, 18 DAT.

Plants were harvested at 19 DAT. Shoots were removed above the collar and divided into leaves, stems, and roots. Fresh masses were recorded, the separated parts were dried in an oven at 70°C for 48 h, and the dry masses were measured to the nearest 0.01 g. The gathering of root architectural data including taproot length, lateral root length, total root length, root system depth and width, and convex hull area has been described previously (Belachew et al., 2018).

Root mass fraction was calculated by dividing root dry mass (g) by total dry mass (g) (root + leaf + stem dry mass). Apparent specific root length (m/g) was calculated by dividing the total root length visible at the transparent plate of the rhizotrons (m) by the root dry mass (g). The term “apparent” is used because the measure was based on the visible length rather than the true length. Apparent enclosed root volume (cm^3^) was calculated by multiplying the convex hull area enclosed in the rhizotrons (cm^2^) by the width of the box (0.050 m), under the assumption that the convex hull area was representative of the true enclosed root volume. Apparent root length density (cm/cm^3^) was calculated by dividing the total visible root length (cm) by the apparent enclosed root volume (cm^3^). Leaf dry matter content was taken as leaf dry mass (g) divided by leaf fresh mass (g) multiplied by 100. Stem dry matter content was taken as stem dry mass (g) divided by stem fresh mass (g) multiplied by 100. Data for total visible root length and root convex hull area were taken from Belachew et al. (2018) to calculate apparent specific root length and apparent root length density. Root architectural data from Belachew et al. (2018) were used in correlation analysis with shoot and root morphological and physiological data reported here. For details, **Supplemental Tables 1** and **2** present the raw data of this study.

### Data Analysis

Data were subjected to analysis of variance using SPSS version 24.0 (IBM Inc., Chicago, IL, USA). Where the error variance was not uniformly distributed between treatments and was proportional to the mean, data were transformed to the normal logarithm for further analysis. Since the transformation neither altered the statistical conclusions nor clarified the significant differences between means, the arithmetic results are reported. Treatment means were separated a posteriori by the least significant difference test (5%). Genotypic means (across treatments) and phenotypic means were both tested. Two-tailed Pearson correlations were calculated, and principal component analyses between root and shoot measurement data were performed. In order to identify the most important correlating variables that maximized variances between treatments and among accessions, and group accessions based on response trait homogeneity, we conducted principal component analysis (PCA) and hierarchical cluster analysis of root and shoot data to construct a dendrogram using average linkage between groups. Correlation network of traits was calculated using R (R Core Team, 2018).

## Results

**Figure 1:**
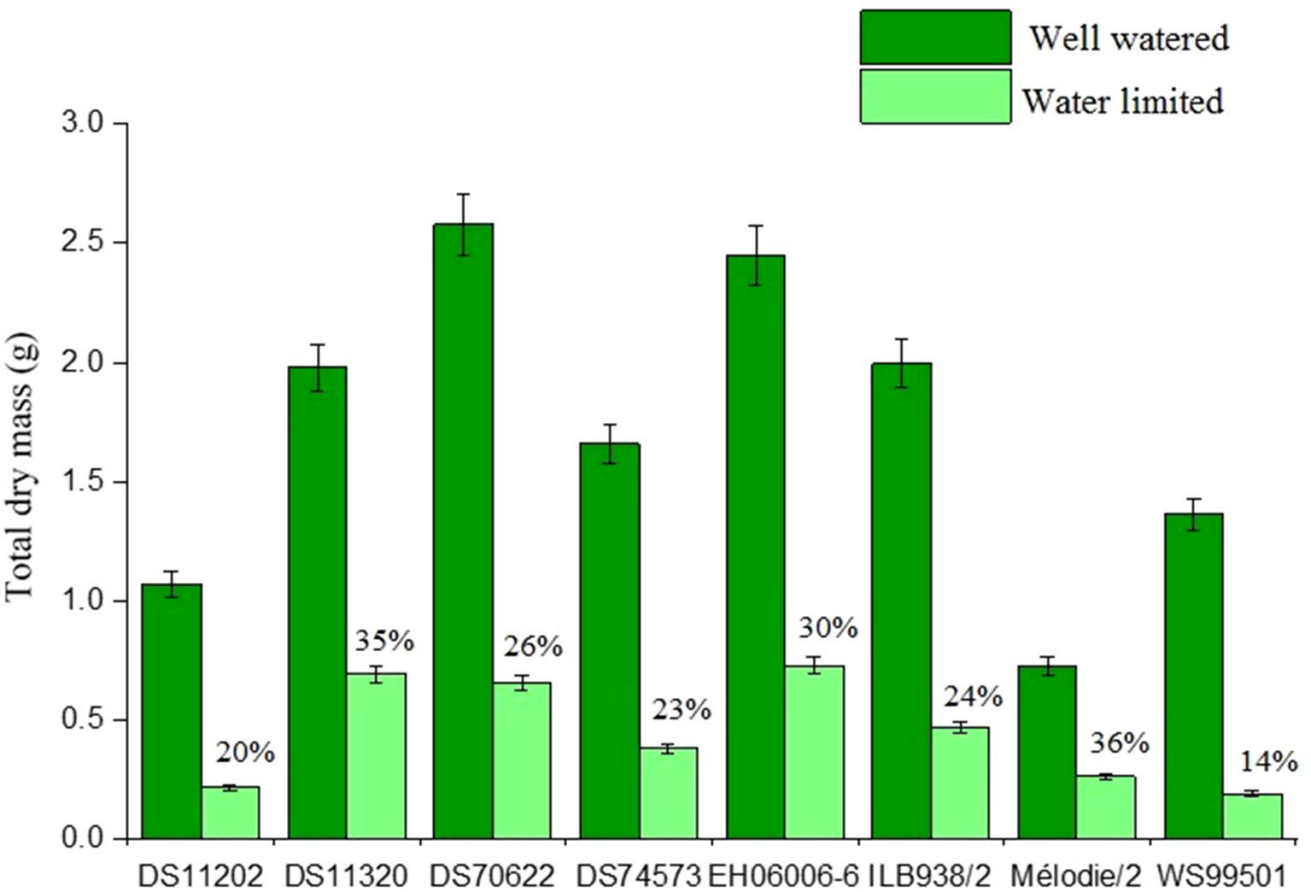
Total dry mass (g) values of eight faba bean accessions. Numbers on top of water-limited bars indicate % biomass accumulation of water-limited plants relative to well-watered plants.

Total, leaf, stem, and root dry masses were all three to four times greater in the well-watered treatment than those in the water-limited one (**Figure 1**, **Supplemental Figure 1**). The watering treatment showed no significant effects on apparent specific root length and apparent root length density (**Table 1**). Leaf dry matter content (well watered = 9.8, water limited = 12.7) and stem dry matter content (**Table 2**) were significantly higher in the water-limited plants, showing that the water deficit treatment was sufficiently severe. Stomatal conductance was 3.8-fold lower in the water deficit treatment than that in the well-watered treatment (**Table 2**), indicating closure of stomata in response to the stress, whereas SPAD value was 1.4-fold higher (P < 0.001) in the water-limited treatment, which may be associated with reduction of leaf size (leaf count and leaf dry mass) (**Table 2** and **Supplemental Figure 1**).

**Table 2:** Leaf number, stomatal conductance, chlorophyll fluorescence, and stem dry matter content of eight faba bean accessions in well-watered and water-limited conditions 28 days after seed imbibition, four biological replicates. ns is not significant, SE is standard error of the mean, LSD is least significant difference.

Accession	Leaf number		Stomatal conductance (mmol H_2_O.m^−2^.s^−1^)		Effective quantum yield (Φ_PSII_)		Stem dry matter content (%)	
	Well watered	Water limited	Well watered	Water limited	Well watered	Water limited	Well watered	Water limited
DS11202	15.0	6.7	371	85	0.688	0.706	7.6	9.0
DS11320	16.8	10.0	272	70	0.710	0.730	8.6	11.2
DS70622	25.5	10.0	341	85	0.709	0.711	7.8	9.8
DS74573	24.5	10.5	282	73	0.693	0.729	7.1	7.7
EH06006-6	18.5	10.8	288	63	0.699	0.718	8.9	11.1
ILB938/2	14.3	6.8	209	42	0.612	0.671	8.9	11.2
Mélodie/2	11.8	8.7	314	127	0.694	0.716	8.5	5.9
WS99501	11.8	5.3	263	66	0.710	0.714	8.6	7.7
Mean	17.7	8.6	292	77	0.689	0.712	8.2	9.2
SE (accession)	1.4		18		0.014		0.6	
LSD (5%)	3.9		53		0.040		1.8	
SE (treatments)	0.7		9		0.007		0.3	
LSD (5%)	1.9		26		ns		0.9	
**P-value**								
Treatment	0.003		0.001		0.103		0.018	
Accession	< 0.001		0.005		0.008		0.005	
Treatment x Accession	0.058		0.422		0.874		0.056	

**Figure 2:**
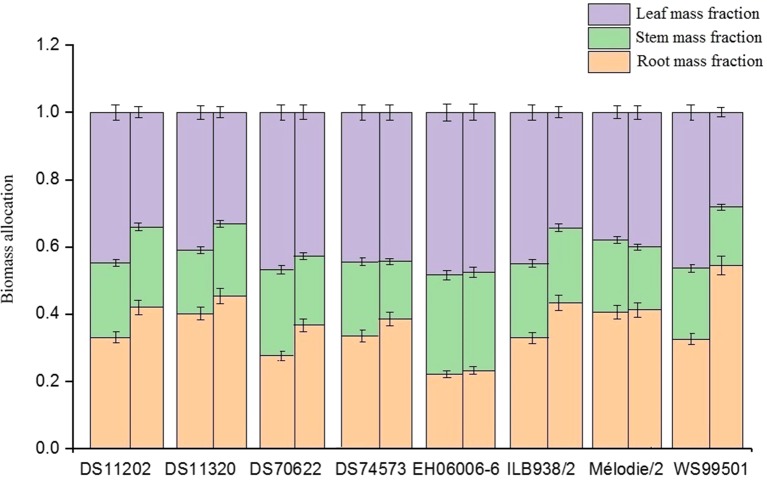
Biomass allocation to root, stem, and leaf mass fractions in eight faba bean accessions. The left bar shows the well-watered treatment, and the right side shows the water-limited treatment.

There were significant differences among accessions in total, leaf, root, and shoot dry mass, root mass fraction, apparent specific root length (all P < 0.001), stem dry matter content, stem and leaf mass fraction, stomatal conductance, EQY (all P < 0.01), and apparent root length density (P < 0.05) (**Tables 1** and **2**, **Figures 1** and **2**, **Supplemental Figure 1**). DS70622 and EH06006-6 produced equally the most dry matter, DS11320 and ILB938/2 about 20% less, and Mélodie/2 the least (**Figure 1**). Stem dry matter content was equally highest in EH06006-6, ILB938/2, and DS11320, intermediate in DS70622, DS11202, and WS99501, and low in DS74573 and Mélodie/2 (**Table 2**). Leaf numbers were equally high in DS70622 and DS74573, followed by EH06006-6 with about 20% less, and WS99501 had the fewest (**Table 2**). Stomatal conductance followed a different pattern, with high values in DS11202, DS70622, and Mélodie 2, intermediate values in DS11320, DS74573, EH06006-6, and WS99501, and the lowest value in ILB938/2 (**Table 2**).

Accessions responded differently to the watering treatments, with significant treatment by accession interactions in total dry mass, shoot dry mass, leaf dry mass (all P < 0.001), stem dry mass (P < 0.01), root dry mass, and apparent root length density (both P < 0.05) (**Table 1**, **Figure 1**, **Supplemental Figure 1**) but not leaf mass fraction or stem mass fraction. In the water-limited treatment, accession DS70622 and DS11320 showed high values of total, root, and shoot dry mass, while EH06006-6 was high in total and shoot dry mass but low in root dry mass. Total dry mass, shoot dry mass, and leaf mass fraction in the well-watered treatment along with apparent specific root length and apparent root length density in both treatments were greater in DS70622 than those in DS11320, which in turn exhibited the larger root mass fraction but lower apparent specific root length and apparent root length density in both treatments (**Table 1**, **Figures 1** and **2**). ILB938/2 performed similarly to DS11320 in these traits. DS70622 produced finer and more densely growing roots than DS11320. EH06006-6 was distinguished by its maintenance of a high stem and leaf mass fraction in the water-limited treatment (**Figure 2**). In the water-limited treatment, Mélodie/2 and WS99501 showed consistently low values of total dry mass, root and shoot dry mass, and apparent root length density (**Table 1**, **Figure 1**, and **Supplemental Figure 1**). DS70622 and DS11202 showed high values of stomatal conductance in the well-watered treatment, whereas Mélodie/2 showed the highest value in the water-limited treatment and ILB938/2 had the lowest values in both treatments.

### Associations Between Traits

**Figure 3:**
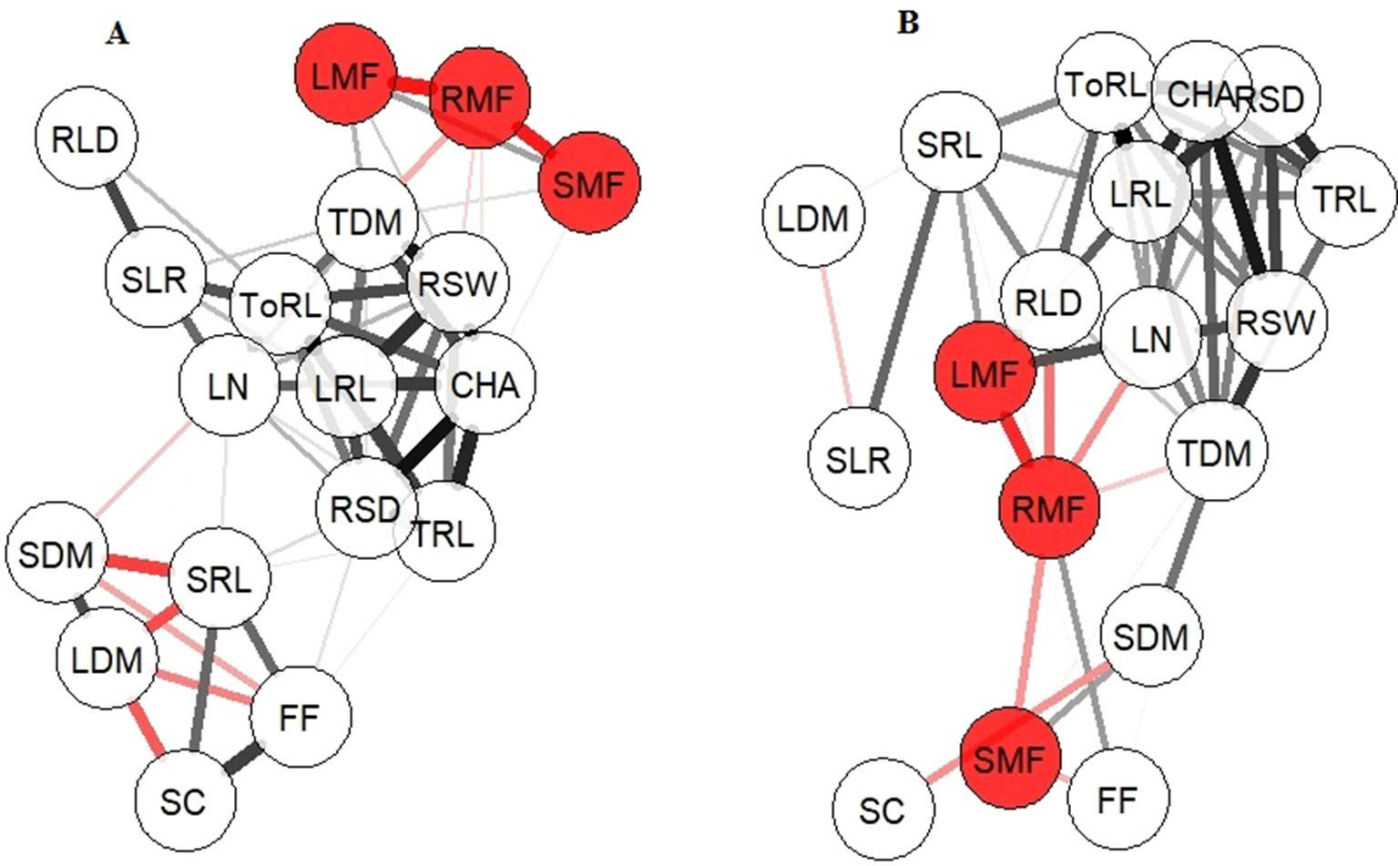
Correlation network for 17 traits. **(A)** Well-watered treatment, **(B)** water-limited treatment. Colored circles indicate root, stem, and leaf mass fractions. Lines in black indicate positive correlations, lines in red indicate negative correlations. Thick lines, |r| > 0.834 (P < 0.01); intermediate lines, 0.720 < |r| < 0.834 (P < 0.05); thin lines, 0.500 < |r| < 0.720 (ns); no line, r < 0.5. Abbreviations: CHA, convex hull area; FF, Fv/Fm; LN, leaf number; LDM, leaf dry matter content; LMF, leaf mass fraction; LN, leaf number; LRL, lateral root length; RLD, apparent root length density; RMF, root mass fraction; RSD, root system depth; RSW, root system width; SC, stomatal conductance; SDM, stem dry matter content; SLR, second order lateral root length; SMF, stem mass fraction; SRL, apparent specific root length; TDM, total dry mass; ToRL, total root length; TRL, taproot length.

In the well-watered treatment, total dry mass was significantly (0.743 ≤ r ≤ 0.967; n = 8) and positively correlated with several root parameters, including lateral and total root length, root system width, and convex hull area, indicating the strong association of a prolific root system with biomass accumulation (**Figure 3A**). Leaf number was correlated with lateral root length and total root length. Stomatal conductance was correlated with apparent specific root length, indicating the association of stomatal opening and thinner roots. Second-order lateral root length was correlated with apparent root length density, indicating the role of fine roots in filling the soil volume. Fv/Fm was correlated with stomatal conductance and apparent specific root length. Significant negative correlations (-0.916 ≤ r ≤ -0.801; n = 8) were found between root mass fraction and both leaf and stem mass fractions, between leaf dry matter content and apparent specific root length, Fv/Fm, and stomatal conductance, and between stem dry matter content and apparent specific root length. These correlations indicate that high specific root length was associated with the maintenance of plant water status in moist conditions.

In the water-limited treatment, total dry mass was again significantly (0.718 ≤ r ≤ 0.998; n = 8) and positively correlated with root traits including root system depth, root system width, and convex hull area as well as with stem dry matter content, indicating the strong association of root depth and width traits with total biomass accumulation (**Figure 3B**). Apparent root length density was correlated with apparent specific root length and total root length, indicating the association of dense root system with whole root system length. Negative correlations (-0.906 ≤ r ≤ -0.709; n = 8) were found between root mass fraction and both leaf and stem mass fraction and root length density, and between stomatal conductance and stem dry matter content.

In summary, total dry mass in the well-watered treatment was strongly associated with root length traits, but in the water limited treatment, it was associated with root system depth, width, and convex hull area. Apparent root length density in the well-watered treatment showed a strong association with fine parts of the root system, namely second-order lateral root length, whereas in the water-limited treatment, its notable association was with apparent specific root length.

### Principal Component and Cluster Analyses

**Figure 4:**
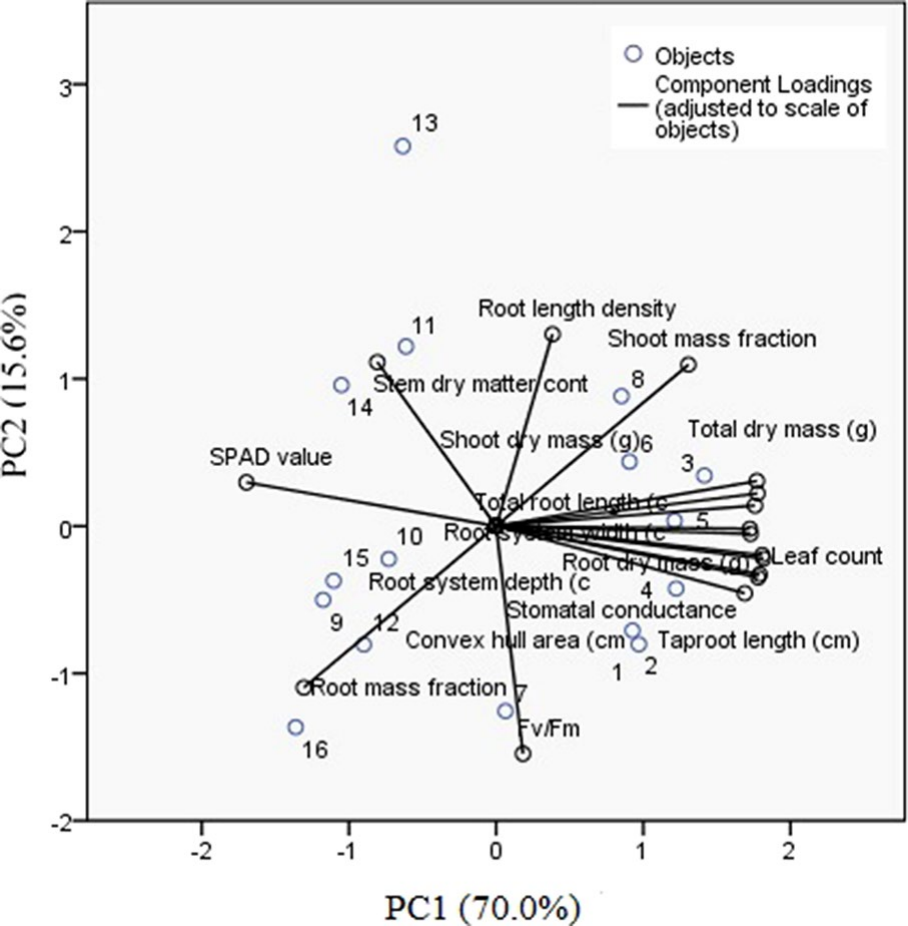
PCA of root and shoot traits for eight faba bean accessions tested in well-watered and water-limited treatments. Numbers 1–8 indicate the accessions in the same order as in **Table 1** in the well-watered treatment and 9–16 in the water-limited treatment.

**Figure 5:**
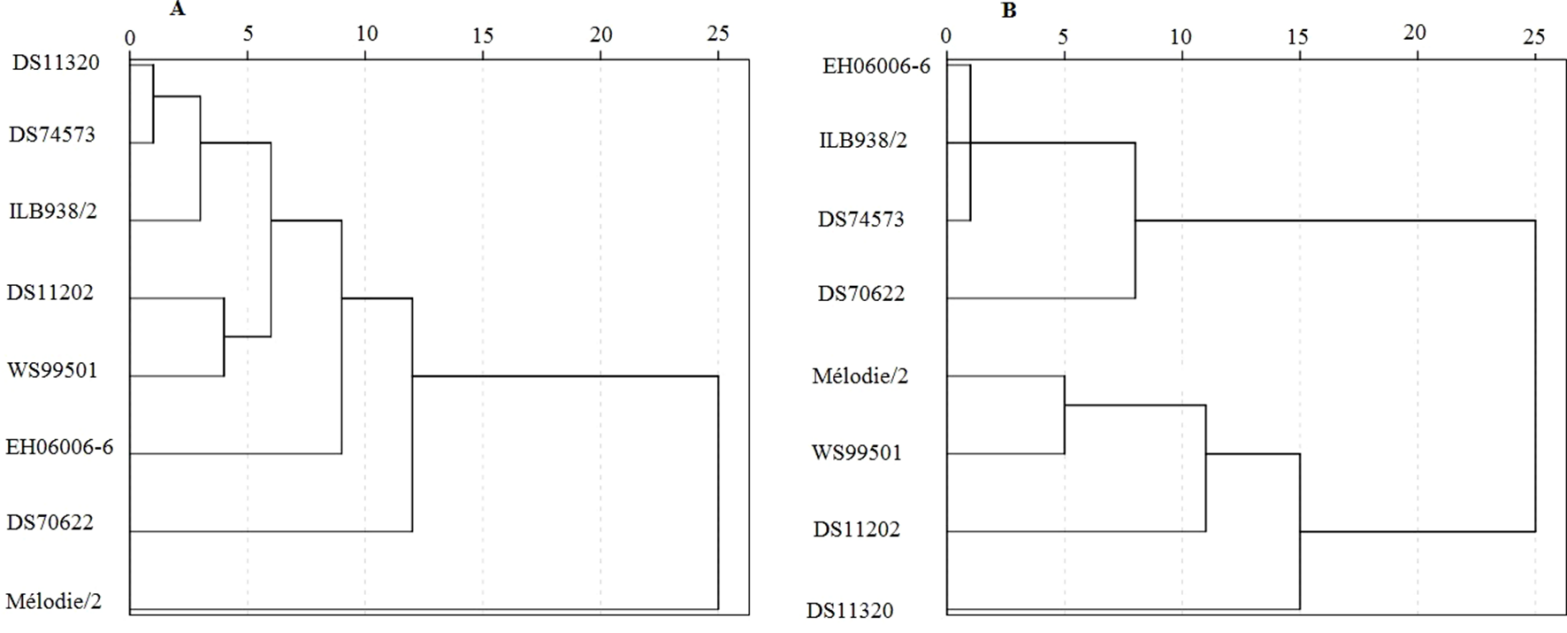
Dendrogram indicating average linkage between groups of eight faba bean accessions. **(A)** Well-watered treatment, **(B)** water-limited treatment. Trait predictors used to construct the linkage were total dry mass, leaf number, leaf dry matter content, stem dry matter content, root mass fraction, stem mass fraction, leaf mass fraction, leaf number, stomatal conductance, Fv/Fm, taproot length, lateral root length, second order lateral root length, total root length, root system depth, root system width, convex hull area, apparent specific root length, and apparent root length density.

The PCA showed a total of 85.6% of the variance in the first two dimensions (**Table 3**). Most of the separation between treatments was achieved by PC1 and that between accessions by PC2 (**Figure 4**). PC1 accounted for 70.0% of the total variance observed and was based primarily on root system size traits including length, depth and area, shoot and root dry masses, stomatal conductance, and SPAD value. PC2 accounted for 15.6% of the total variance observed and was based primarily on Fv/Fm, apparent root length density, and root mass fraction. Cluster analysis confirmed that DS70622 was well distinguished from all the other accessions in both well-watered and water-limited conditions (**Figure 5**). In the well-watered treatment, Mélodie/2 was an outlier from the rest, and EH06006-6 was moderately separated from the cluster that contained the remaining five accessions. In the water-limited treatment, three accessions formed a cluster associated with DS70622, while the other cluster contained another three accessions loosely associated with DS11320.

**Table 3:** Trait characteristics of 8 faba bean accessions in well-watered and water limited treatments in the PCA and the eigenvectors.

Traits	Total (Vector Coordinates)		
	PC1	PC2	Total
Convex hull area (cm^2^)	**0.97**	0.01	0.98
Leaf count	**0.96**	0.01	0.97
Root system depth (cm)	**0.94**	0.03	0.97
Taproot length (cm)	**0.93**	0.04	0.96
Shoot dry mass (g)	**0.92**	0.01	0.94
Total dry mass (g)	**0.92**	0.03	0.95
Total root length (cm)	**0.91**	0.01	0.91
Root system width (cm)	**0.88**	0.00	0.88
Root dry mass (g)	**0.87**	0.00	0.87
SPAD value	**0.84**	0.03	0.87
Stomatal conductance	**0.84**	0.06	0.90
Shoot mass fraction	**0.50**	0.35	0.85
Root mass fraction	**0.50**	0.35	0.85
Fv/Fm	0.01	**0.70**	0.71
Apparent root length density (cm/cm^3^)	0.04	**0.50**	0.54
Stem dry matter content (%)	0.19	**0.36**	0.55
% of Variance	70.02	15.56	85.58

The largest variable loading scores across the two principal components are shown in bold.

## Discussion

Drought treatment resulted in the reduction of shoot mass, root mass, and stomatal conductance but increases in leaf and stem dry matter content and SPAD values. Data from root and shoot measurements indicated strong associations, and root traits contributing to drought avoidance were positively correlated with shoot traits. Accessions showed important differences in their responses to water deficit, with the maintenance of dry mass and apparent root length density along with reduced stomatal conductance under stress conditions being particularly informative.

The negative correlation of root mass fraction with apparent root length density and stem and leaf mass fraction under water-limited treatment (**Figure 3B**) can be explained by positive feedback between root exploration of the soil volume, finding water that maintains shoot growth that in turn continues to provide assimilate for root growth. Hence, root mass fraction in water-limited conditions indicated two different outcomes. In DS11320 and ILB938/2, it indicated their ability to maintain a substantial energy allocation to the root system under moisture deficiency (**Figure 2** and **Supplemental Figure 1**). Increased root mass fraction was reported to increase water absorption in alfalfa under severe drought (Erice et al., 2010). As discussed by Blum (1996), the increase in root mass fraction in water-limited conditions has implications in the maintenance of plant water status when transpiration reaches critical levels, and the continuation of root growth and the corresponding arrest in shoot growth were controlled by abscisic acid (ABA) accumulation in roots and OA in shoots in response to water deficit. In faba bean, risk-taking by minimal reduction of shoot dry mass under water stress was reported as a physiological indicator for drought tolerance at the flowering stage (Khan et al., 2007). In soybean, the capacity of genotypes to sustain shoot biomass in both water and nitrogen deficiency was shown to be an important trait for drought tolerance (Fenta et al., 2012). In Mélodie/2, WS99501, and DS11202, however, the observed high root mass fraction was associated with poor shoot development under water limitation (**Table 1**, **Figure 2**).

Accessions DS70622, DS11320, and ILB938/2 showed relatively high values of total, root and shoot dry mass, and low values of stomatal conductance in the water-limited treatment, but they differed in many other aspects. In both treatments, DS70622 showed high apparent specific root length and apparent root length density in roots that were distributed well in depth and width together with the second highest values of stomatal conductance, whereas DS11320 and ILB938/2 showed higher root mass fraction and lower apparent specific root length and apparent root length density. Consequently, they exhibited different drought avoidance mechanisms. DS70622 maximized its water search and absorption potential by producing finer roots (high apparent specific root length) with higher apparent root length density, thereby exploring the root volume comprehensively, demonstrated by its relatively high stomatal conductance in the water-limited treatment, and leading to its outlying position in the cluster analysis. DS11320 enclosed almost as large a soil volume as DS70622 but filled the volume less well, with larger and thicker roots, and had relatively low stomatal conductance in both treatments, showing that this strategy apparently did not find adequate amounts of water. ILB938/2 showed the lowest stomatal conductance, apparent specific root length, and apparent root length density in both treatments, suggesting that its drought tolerance is based on closed stomata rather than accessing water. Low stomatal conductance in ILB938/2 has previously been interpreted as an indicator of drought tolerance (Khan et al., 2007; Khazaei et al., 2018), but the ability of the plant to regulate stomatal conductance may be a better strategy than continuously low conductance, as shown in common bean (*Phaseolus vulgaris* L. cv. “Pinto Saltillo”) (Rosales et al., 2012). Mélodie/2 combined low total dry mass, specific root length, apparent root length density, and high stomatal conductance in both treatments, suggesting that it may be sensitive to limitation of water in the vegetative phase, in contrast to previous results obtained on plants at the flowering stage (Khan et al., 2007; Khazaei et al., 2013b). These results suggest that it is the ability to access a larger soil volume, as shown by DS70622, that best maintains total dry matter production in water-limited conditions and that this cannot be detected only in well-watered conditions. When water was not limited, total dry matter was correlated with root-length traits, suggesting that the whole plant was growing well.

Differences between accessions in the drought response of root mass fraction fell into three classes, namely, no, moderate, and severe responses (**Figure 2**). Plants subjected to moderate drought may respond to the stress with moderate or no change to root mass fraction as compared with control plants, but those plants exposed to severe drought may lose 50% of their total biomass (Poorter et al., 2012). By these criteria, EH06006-6 and Mélodie/2 showed very little response to the water deficit and WS99501 responded as if severely water-limited, whereas DS11202, DS11320, DS70622, and DS74573 were apparently responding appropriately to the stress.

A further approach is to test the fit to isohydric (water-saving) and anisohydric (water-spending) models of adaptation to drought (Blum, 2015). In isohydric plants, drought signals such as drying soil, low leaf water potential, or high air vapor pressure deficit induce the biosynthesis of ABA in roots or leaves, which triggers closure of stomata, arrests growth, and allows homeostasis of leaf water potential by increased uptake of water from the soil until the water potential gradient between the plant cells and the soil is reversed. In contrast, anisohydric plants in the same conditions exhibit little increase in ABA content but greater OA, producing prolific roots for effective soil water extraction, maintaining leaf water potential and gas exchange, ultimately leading to acceptable growth and productivity. Taking total dry mass, stomatal conductance, and root traits as criteria, WS99501, DS11320, ILB938/2, and DS74573 conform to the isohydric model, while DS70622 and EH06006-6 fit the anisohydric model. DS11202 and Mélodie/2 fall between the models, and it is possible that they enhance both ABA and OA mechanisms to maintain their leaf water potential. The anisohydric model provides an opportunity to identify sources of adaptation to transient drought that can be used in practical breeding, particularly when the underlying quantitative trait loci and eventually genes are characterized for use in marker-assisted selection. Similarly, where drought is expected to be severe, a fit to the isohydric model can be taken as an indication of breeding potential.

Significantly higher values of SPAD and EQY and lower values of stomatal conductance were observed in the water-limited treatment. Accessions with large root systems, such as DS70622 and DS11320 (but not ILB938/2), showed higher values of EQY (**Table 2**), suggesting that they apparently avoided the drought. Higher SPAD values were associated with reduced leaf dry mass and leaf count and increased leaf dry matter content, showing that the chlorophyll was more concentrated in the smaller leaves, as shown previously (Khazaei et al., 2013b). Although leaf expansion is limited at the beginning of a drought episode by lack of turgor, the plant often maintains carbon assimilation near the normal level (Blum, 1996). In terminal drought conditions, however, the opposite occurs, as shown in lentil (Kumar et al., 2012; Idrissi et al., 2015).

There were significant differences (P < 0.01) between treatment means of Fv/Fm, but the values recorded were in the range of expected optima for many plant species, which is between 0.79 and 0.84 (Maxwell and Johnson, 2000). Fenta et al. (2012) reported Fv/Fm values as low as 0.38 in young plants of two soybean cultivars within 8 days of water stress, showing the photo-inhibitory characteristics of water deficit. The minimum effect of different soil moisture levels on photosynthetic traits including chlorophyll content and Fv/Fm was observed in drought-tolerant genotypes of barley but with variation at different stages of growth, with Fv/Fm values of 0.79 for the control and 0.73 for plants water-limited (Li et al., 2006).

## Conclusion

Drought treatment of faba bean in the first 3 weeks of growth resulted in the expected reductions in biomass and stomatal conductance and in increases in SPAD value. Apparent specific root length, apparent root length density, root and stem mass fraction, and EQY showed strong differences between accessions. The accession by treatment interactions in total dry mass and apparent root length density was significant, indicating that some accessions were more tolerant than others. Thus, the interaction of several traits has to be examined in order to identify drought-avoiding accessions.

Accessions showed contrasting drought-avoidance mechanisms. In contrast to previous results obtained at the flowering stage, Mélodie/2 was found to be sensitive to water deficit in the vegetative stage of growth. DS70622 combined high apparent specific root length with high apparent root length density, thus maximizing its water-absorption capacity with dense and fine roots growing well in depth and width in both treatments. This accession is thus a suitable source of traits for adaptation to transient drought conditions. DS11320 explored a comparable root volume with lower apparent root length density and apparent specific root length but did not find enough water to maintain its stomatal conductance. ILB938/2 maximized its moisture content by closing its stomata and not by any analyzed aspect of its root system. Hence, DS11320 and ILB938/2 are sources of adaptation to severe drought conditions. The root and shoot morphological plasticity shown by these diverse accessions may be related with their adaptations to water availability in their original habitats, namely Syria, Macedonia, and Ecuador.

## Author Contributions

KB, FS, and KN contributed conception and design of the study; KB conducted the experiment and organized the database; KB, FS, and HP performed the statistical analysis; KB wrote the first draft of the manuscript; FS, HP, KN, and KB wrote the sections of the manuscript; all authors contributed to manuscript revision and read and approved the submitted version.

## Funding

Finnish Cultural Foundation: 0116947-3 covered the working grant of the PI. COST Action: FA1306 covered travel grant of the researcher during the conduct of the research. Open access publication fee was covered by University of Helsinki.

## Conflict of Interest Statement

KN and HP are employees of Forschungszentrum Jülich GmbH, Germany. The remaining authors declare that the research was conducted in the absence of any commercial or financial relationship that could be construed as potential conflict of interest.

## References

[B1] Arunyanark A. Jogloy S. Akkasaeng C. Vorasoot N. Kesmala T. Rao R. C. N. (2008). Chlorophyll stability is an indicator of drought tolerance in peanut. J. Agron. Crop Sci. 194, 113–125. 10.1111/j.1439-037X.2008.00299.x

[B2] Belachew K. Y. Nagel A. K. Fiorani F. Stoddard F. L. (2018). Diversity in root growth responses to moisture deficit in young faba bean (*Vicia faba L.*) plants. PeerJ 6, 1–20. 10.7717/peerj.4401 PMC5826991 29492343

[B3] Belachew K. Y. Stoddard F. L. (2017). Screening of faba bean (*Vicia faba L.*) accessions to acidity and aluminium stresses. PeerJ 5, 1–19. 10.7717/peerj.2963 PMC5301972 28194315

[B4] Blum A. (1996). Crop responses to drought and the interpretation of adaptation. J. Plant Growth Regul. 20, 135–148. 10.1007/BF00024010

[B5] Blum A. (2009). Effective use of water (EUW) and not water-use efficiency (WUE) is the target of crop yield improvement under drought stress. Field Crops Res. 112, 119–123. 10.1016/j.fcr.2009.03.009

[B6] Blum A. (2015). Towards a conceptual ABA ideotype in plant breeding for water limited environments. Funct. Plant Biol. 42, 502–513. 10.1071/FP14334 32480696

[B7] Coleman J. S. McConnaughay K. D. M. Ackerly D. D. (1994). Interpreting phenotypic variation in plants. Trends Ecol. Evol. 9, 187–191. 10.1016/0169-5347(94)90087-6 21236817

[B8] Duc G. Aleksić J. M. Marget P. Mikić A. Paull J. Redden R. J., (2015). “Faba bean,” in Grain Legumes: Handbook of Plant Breeding 10. De Ron AM, editor. New York: Springer Science. 10.1007/978-1-4939-2797-5_5

[B9] Erice G. Louahlia S. Irigoyen J. J. Sanchez-Diaz M. Avice J.-C. (2010). Biomass partitioning, morphology and water status of four alfalfa genotypes submitted to progressive drought and subsequent recovery. J. Plant Physiol. 167, 114–120. 10.1016/j.jplph.2009.07.016 19744745

[B10] Farooq M. Gogoi N. Barthakur S. Baroowa B. Bharadwaj N. Alghamdi (2017). Drought stress in grain legumes during reproduction and grain filling. J. Agron. Crop Sci. 203, 81–102. 10.1111/jac.12169

[B11] Fenta B. A. Driscoll S. P. Kunert K. J. Foyer C. H. (2012). Characterization of drought-tolerance traits in nodulated soya beans: The importance of maintaining photosynthesis and shoot biomass under drought-induced limitations on nitrogen metabolism. J. Agron. Crop Sci. 198, 92–103. 10.1111/j.1439-037X.2011.00491.x

[B12] Gogoi N. Farooq M. Barthakur S. Baroowa B. Paul S. Bharadwaj N. (2018). Thermal stress impacts on reproductive development and grain yield in grain legumes. J. Plant Biol. 61, 265–291. 10.1007/s12374-018-0130-7

[B13] Gosa C. S. Lupo Y. Moshelion M. (2018). Quantitative and comparative analysis of whole-plant performance for functional physiological traits phenotyping: new tools to support pre-breeding and plant stress physiology studies. Plant Sci. 282, 49–59. 10.1016/j.plantsci.2018.05.008 31003611

[B14] Halperin O. Gebremedhin A. Wallach R. Moshelion M. (2016). High-throughput physiological phenotyping and screening system for the characterization of plant-environment interactions. Plant J. 89, 839–850. 10.1111/tpj.13425 27868265

[B15] Idrissi O. Houasli C. Udupa S. M. De Keyser E. Van Damme P. De Riek J. (2015). Genetic variability for root and shoot traits in a lentil (*Lens culinaris Medik.*) recombinant inbred line population and their association with drought tolerance. Euphytica 204, 693–709. 10.1007/s10681-015-1373-8

[B16] Khan H. R. Link W. Hocking T. J. Stoddard F. L. (2007). Evaluation of physiological traits for improving drought tolerance in faba bean (*Vicia faba L.*). Plant Soil 292, 205–217. 10.1007/s11104-007-9217-5

[B17] Khan H. R. Paull J. G. Siddique K. H. M. Stoddard F. L. (2010). Faba bean breeding for drought-affected environments: a physiological and agronomic perspective. Field Crops Res. 115, 279–286. 10.1016/j.fcr.2009.09.003

[B18] Khazaei H. Link W. Street K. Stoddard F. L. (2018). ILB938, a valuable faba bean accession. Plant Genet. Resour. 16, 478–482. 10.1017/S1479262118000205

[B19] Khazaei H. Street K. Bari A. Mackay M. Stoddard F. L. (2013a). The FIGS (Focused Identification of Germplasm Strategy) approach identifies traits related to drought adaptation in *Vicia faba* genetic resources. PLoS ONE 8, e63107. 10.1371/journal.pone.0063107 23667581 PMC3648475

[B20] Khazaei H. Street K. Santanen A. Bari A. Stoddard F. L. (2013b). Do faba bean (*Vicia faba L.*) accessions from environments with contrasting seasonal moisture availabilities differ in stomatal characteristics and related traits. Genet. Resour. Crop Evol. 60, 2343–2357. 10.1007/s10722-013-0002-4

[B21] Krishnamurthy L. Kashiwagi J. Gaur P. M. Upadhyaya H. D. Vadez V. (2010). Sources of tolerance to terminal drought in the chickpea (*Cicer arietinum L.*) minicore germplasm. Field Crops Res. 119, 322–330. 10.1016/j.fcr.2010.08.002

[B22] Kumar J. A. D. Basu B. P. S. Srivastava E. A. Chaturvedi S. K. A. Nadarajan N. A. Kumar S. C. (2012). Phenotyping of traits imparting drought tolerance in lentil. Crop Pasture Sci. 63, 547–554. 10.1071/CP12168

[B23] Levitt J. (1980). Responses of plants to environmental stresses. Vol. 2. water, radiation, salt and other stresses. New York, USA: Academic Press.

[B24] Li R. Guo P. Baum M. Grando S. Ceccarelli S. (2006). Evaluation of chlorophyll content and fluorescence parameters as indicators of drought tolerance in barley. Agric. Sci. China 5, 751–757. 10.1016/S1671-2927(06)60120-X

[B25] Link W. Hanafy M. Malenica N. Jacobsen H. J. Jelenic S., (2008). “Faba bean,” Compendium of Transgenic Crop Plants, Part 3: Transgenic Legumes Grains and Forages. Kole C Hall TC, editors. Oxford, Uk: Blackwell Publishing Ltd Ch. 4, pp. 71-88. 10.1002/9781405181099.k0304

[B26] Loss S. P. Siddique K. H. M. Martin L. D. (1997a). Adaptation of faba bean (*Vicia faba L.*) to dryland Mediterranean-type environments II. Phenology, canopy development, radiation absorption and biomass partitioning. Field Crops Res. 52, 29–41. 10.1016/S0378-4290(96)03454-5

[B27] Loss S. P. Siddique K. H. M. Tennant D. (1997b). Adaptation of faba bean (*Vicia faba L.*) to dryland Mediterranean-type environments III. Water use and water-use efficiency. Field Crops Res. 54, 153–162. 10.1016/S0378-4290(97)00042-7

[B28] Matsui T. Singh B. B. (2003). Root characteristics in cowpea related to drought tolerance at the seedling stage. Exp. Agr. 39, 29–38. 10.1017/S0014479703001108

[B29] Maxwell K. Johnson N. (2000). Chlorophyll fluorescence – a practical guide. J. Exp. Bot. 51, 659–668. 10.1093/jexbot/51.345.659 10938857

[B30] Mukeshimana G. Lasley A. L. Loescher W. H. Kelly J. D. (2014). Identification of shoot traits related to drought tolerance in common bean seedlings. J. Am. Soc. Hortic. Sci. 139, 299–309. 10.21273/JASHS.139.3.299

[B31] Nagel K. A. Putz A. Gilmer F. Heinz K. Fischbach A. Pfeifer J. (2012). GROWSCREEN-Rhizo is a novel phenotyping robot enabling simultaneous measurements of root and shoot growth for plants grown in soil-filled rhizotrons. Funct. Plant Biol. 39, 891–904. 10.1071/FP12023 32480839

[B32] Negin B. Moshelion M. (2017). The advantages of functional phenotyping in pre-field screening for drought-tolerant crops. Funct. Plant Biol. 44, 107–118. 10.1071/FP16156 32480550

[B33] Passioura J. B. (2012). Phenotyping for drought tolerance in grain crops: when is it useful to breeders? Funct. Plant Biol. 39, 851–859. 10.1071/FP12079 32480835

[B34] Poorter H. Fiorani F. Pieruschka R. Wojciechowski T. van der Putten W. H. Kleyer M. (2016). Pampered inside, pestered outside? Differences and similarities between plants growing in controlled conditions and in the field. New Phytol. 201, 838–855. 10.1111/nph.14243 27783423

[B35] Poorter H. Niklas K. J. Reich P. B. Oleksyn J. Poot P. Mommer L. (2012). Biomass allocation to leaves, stems and roots: meta-analyses of interspecific variation and environmental control. New Phytol. 193, 30–50. 10.1111/j.1469-8137.2011.03952.x 22085245

[B36] Poorter H. Niinemets Ü. Walter A. Fiorani F. Schurr U. (2010). A method to construct dose–response curves for a wide range of environmental factors and plant traits by means of a meta-analysis of phenotypic data. J. Exp. Bot. 61, 2043–2055. 10.1093/jxb/erp358 20048331

[B37] Purushothaman R. Krishnamurthy L. Deo Upadhyaya H. Vadez V. Varshney R. K. (2016). Shoot traits and their relevance in terminal drought tolerance of chickpea (*Cicer arietinum L.*). Field Crops Res. 197, 10–27. 10.1016/j.fcr.2016.07.016 27698531 PMC5035057

[B38] R Core Team (2018). R: A language and environment for statistical computing.

[B39] Reynolds M. P. Trethowan R. M., (2007). “Physiological interventions in breeding for adaptation to abiotic stress,” in Scale and Complexity in Plant Systems Research: Gene-Plant-Crop Relations. Spiertz JHJ Struik PC van Laar HH, editors. Dordrecht, Netherlands: Springer, 129–146. 10.1007/1-4020-5906-X_11

[B40] Rosales M. A. Ocampo E. Rodríguez-Valentín R. Olvera-Carrillo Y. Acosta-Gallegos J. Covarrubias A. A. (2012). Physiological analysis of common bean (*Phaseolus vulgaris L.*) cultivars uncovers characteristics related to terminal drought resistance. Plant Physiol. Biochem. 56, 24–34. 10.1016/j.plaphy.2012.04.007 22579941

[B41] Sarker A. Erskine W. Singh M. (2005). Variation in shoot and root characteristics and their association with drought tolerance in lentil landraces. Genet. Resour. Crop Evol. 52, 89–97. 10.1007/s10722-005-0289-x

[B42] Singh D. Dikshit H. K. Singh R. (2013). A new phenotyping technique for screening for drought tolerance in lentil (*Lens culinaris Medik.*). Plant Breed 132, 185–190. 10.1111/pbr.12033

[B43] Stoddard F. L. Balko C. Erskine W. Khan H. R. Link W. Sarker A. (2006). Screening techniques and sources of resistance to abiotic stresses in cool-season food legumes. Euphytica 147, 167–186. 10.1007/s10681-006-4723-8

[B44] Tuberosa R. (2012). Phenotyping for drought tolerance of crops in the genomics era. Front. Physiol. 3, 347. 10.3389/fphys.2012.00347 23049510 PMC3446691

[B45] Ye H. Roorkiwal M. Valliyodan B. Zhou L. Chen P. Varshney R. K. (2018). Genetic diversity of root system architecture in response to drought stress in grain legumes. J. Exp. Bot. 69, 3267–3277. 10.1093/jxb/ery082 29522207

[B46] Zhao J. Sykacek P. Bodner G. Rewald B. (2017). Root traits of European *Vicia faba* cultivars-using machine learning to explore adaptations to agronomic conditions. Plant Cell Environ. 41, 1984–1986. 10.1111/pce.13062 28857245

[B47] Zhou R. Hyldgaard B. Yu X. Rosenquist E. Ugarte R. M. Yu S. (2018). Phenotyping of faba beans (*Vicia faba L.*) under cold and heat stresses using chlorophyll fluorescence. Euphytica 214, 68. 10.1007/s10681-018-2154-y

