# Supplementary figures and images for: Association of Shoot and Root Responses to Water Deficit in Young Faba Bean (Vicia faba L.) Plants
Source: Front Plant Sci. 2019 Sep 4;10:1063. doi: 10.3389/fpls.2019.01063 (PMC6738164; doi:10.3389/fpls.2019.01063)

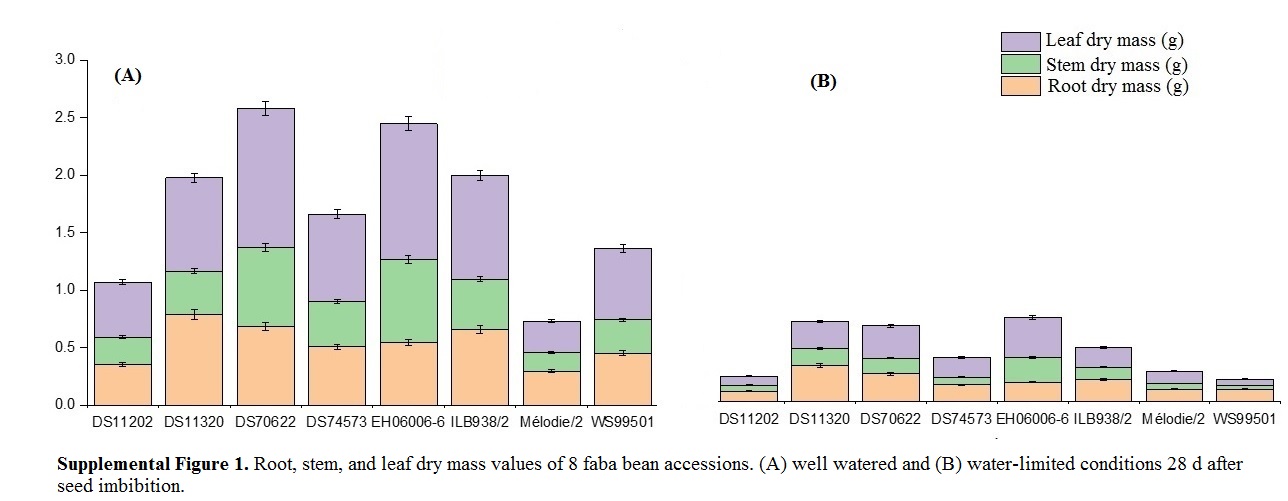

Supplement: Supplementary file 3 [file Image_1.jpg]
